# Neighbourhood socioeconomic conditions and emergency admissions for ambulatory care sensitive conditions in children: a longitudinal ecological analysis in England, 2012–2017

**DOI:** 10.1136/bmjpo-2024-002991

**Published:** 2025-01-19

**Authors:** Courtney Franklin, Kate Mason, Lateef Akanni, Konstantinos Daras, Tanith Rose, Bernie Carter, Enitan D Carrol, David Taylor-Robinson

**Affiliations:** 1 Institute of Population Health, University of Liverpool Faculty of Health and Life Sciences, Liverpool, UK; 2 The University of Melbourne School of Population and Global Health, Melbourne, Victoria, Australia; 3 NIHR Health Protection Research Unit in Gastrointestinal Infections, Liverpool, UK; 4 Department of Public Health and Policy, University of Liverpool, Liverpool, UK; 5 Edge Hill University Faculty of Health and Social Care, Ormskirk, UK; 6 Institute of Infection and Global Health, University of Liverpool, Liverpool, UK

**Keywords:** Child Health, Health services research, Epidemiology

## Abstract

**Background:**

Ambulatory care sensitive conditions (ACSCs) are those for which hospital admission could be prevented by interventions in primary care. Children living in socioeconomic disadvantage have higher rates of emergency admissions for ACSCs than their more affluent counterparts. Emergency admissions for ACSCs have been increasing, but few studies have assessed how changing socioeconomic conditions (SECs) have impacted this. This study investigates the association between local SECs and emergency ACS hospital admissions in children in England.

**Methods:**

We examined longitudinal trends in emergency admission rates for ACSCs and investigate the association between local SECs and these admissions in children over time in England, using time-varying neighbourhood unemployment as a proxy for SECs. Fixed-effect regression models assessed the relationship between changes in neighbourhood unemployment and admission rates, controlling for unmeasured time-invariant confounding of each neighbourhood. We also explore the extent to which this relationship differs by acute and chronic ACSCs and is explained by access to primary and secondary care.

**Results:**

Between 2012 and 2017, paediatric emergency admissions for acute ACSCs increased, while admissions for chronic ACSCs decreased. At the neighbourhood level, each 1% point increase in unemployment was associated with a 3.9% and 2.7% increase in the rate of emergency admissions for acute ACSCs, for children aged 0–9 years and 10–19 years, respectively. A 2.6% increase in admission rates for chronic ACSCs was observed, driven by an association in 0–9 years old. Adjustment for primary and secondary care access did not meaningfully attenuate the magnitude of this association.

**Conclusions:**

Increasing trends in neighbourhood unemployment were associated with increases in paediatric emergency admission rates for ACSCs in England. This was not explained by available measures of differential access to care, suggesting policy interventions should address the causes of unemployment and poverty in addition to health system factors to reduce emergency admissions for ACSCs.

**WHAT IS ALREADY KNOWN ON THIS TOPIC**

- Children living in disadvantaged socioeconomic circumstances have a higher risk of emergency hospital admissions than their more affluent counterparts.
- Emergency admissions for conditions that might be manageable in primary care have been increasing.

**WHAT THIS STUDY ADDS**

- Emergency hospital admission rates for ambulatory care sensitive conditions (ACSCs) for children increased over time, driven by increasing admissions for acute ACSCs.
- Increasing unemployment was associated with increasing admission rates for both acute and chronic ACSCs in younger children (0–9 years).
- Access to primary and secondary care measures did not meaningfully attenuate the association between increasing unemployment and trends in admission rates for ACSCs.

**HOW THIS STUDY MIGHT AFFECT RESEARCH, PRACTICE OR POLICY**

- Improving socioeconomic conditions for children may help reduce emergency admissions for paediatric ACSCs.

## Introduction

 Ambulatory care sensitive conditions (ACSCs), are those conditions for which hospital admission could be prevented by interventions in primary or outpatient care.^1^ ACSCs, avoidable admissions, preventable hospitalisations and avoidable hospitalisations have been used interchangeably in the literature to mean hospitalisations that could have been avoided with timely effective outpatient care (not necessarily primary care).^2^

UK-based studies have reported between 11% and 40% of emergency department (ED) attendances are non-urgent, or ‘inappropriate’.^3 4^ Prior to the COVID-19 pandemic, emergency admissions for ACSCs increased by 18% between 2008 and 2020.^5^ These rates continue to steadily increase,^6 7^ with the proportion of avoidable admissions rising faster than the overall rate of emergency admissions (a 14% increase of emergency admissions for conditions that might be manageable in primary care, compared with a 9.3% increase for all types of emergency admissions).^8^ While emergency admissions for ACSCs can sometime resolve underlying health issues for individuals, collectively they also put significant pressures on the National Health Service (NHS), such as delays in treatment for other patients,^9^ and have been estimated to cost the NHS £1.42 billion annually.^10^ Children admitted to the hospital are also at risk of infections, medical errors, drug reactions and emotional trauma.^11 12^

Children account for a high proportion of ACS admissions, with approximately 14% of all admissions being patients under 5 years old; of these, children are predominantly admitted for acute conditions.^10^ The historic rise in ACS hospitalisations in children has been driven by an increase in acute conditions, while chronic conditions decreased, suggesting justification for dividing ACSCs into chronic and acute conditions.^13 14^ Previous work has also suggested an adult definition for such conditions may be poorly suited to children, and has instead, used a child-centric definition of ACSCs.^14 15^

Individuals living in socioeconomic disadvantage have a higher risk of emergency hospital admissions than their more affluent counterparts.^816^,^21^ A social gradient in emergency admissions for ACSCs has also been established; however, this evidence is limited.^13 19 22^ Ethnicity, age, distance to hospital and access to primary care are also risk factors for avoidable hospital admission.^18 19^ Between 2007 and 2017, divergent patterns of healthcare use along a social gradient were found among children in England; children living in more deprived areas made greater use of emergency services and received less scheduled care than their more affluent counterparts.^23 24^ This inequality gap in ED admissions has remained relatively unchanged over time.^23^

To our knowledge, longitudinal methods have not been used to explore the effect of changing neighbourhood socioeconomic conditions (SECs) on paediatric hospitalisations for ACSCs or explore these differences between chronic and acute ACSCs. This study assesses inequalities in emergency admission rates for ACSCs among children and uses longitudinal methods to investigate the effects of area-level unemployment (as a time-varying measure of neighbourhood deprivation) on emergency ACS admissions for children in England. Specifically, the study will explore the relationship between small-area SECs and hospital admissions for ACSCs in children in England and will help to explain any variation in patterns of these admissions and associated factors between populations or communities.

## Methods

We performed a longitudinal ecological analysis using data collected between 2012 and 2017, across MSOAs (middle super output layers) in England. MSOAs (referred to as neighbourhoods) are geographical areas, made up of groups of LSOAs (lower super output areas), used by the UK Office for National Statistics (ONS), each containing a population between 2000 and 6000 households.^25^ There are currently 6791 MSOAs in England. Outcome data were available between 2008 and 2019. Initial descriptives were explored within this period. Final regression analyses were restricted to 2012–2017 because access to care measures was limited to this time.

### Data

The primary data were obtained from Hospital Episode Statistics, a data warehouse that contains details of clinical, patient, administrative and geographical information about an individual patient admitted to an NHS hospital in England. The primary outcome of interest was emergency admissions (emergency hospital inpatient stays) for ACSCs. We split this outcome into three outcome variables based on ICD-10 (International Classification of Diseases 10th Revision) codes: ED admissions with a primary diagnosis of (1) an acute ACSC (vaccine-preventable diseases, lower respiratory tract infections, upper respiratory tract infections, dehydration and gastroenteritis, urinary tract infections); (2) a chronic ACSC (asthma, diabetes, epilepsy); and (3) any ACSC (all ACS conditions). This approach to defining ACSCs has been used elsewhere.^14^ ICD-10 codes are detailed in supplementary file (online supplemental table S24). We derived biennial emergency admissions for each neighbourhood in England, from 2012 to 2017. Due to the suppression of data, annual data were not available.

The primary exposure variable was the annual neighbourhood unemployment rate, defined as the percentage of people aged 16–64 years in each neighbourhood who were claiming Jobseeker’s Allowance or Universal Credit principally for the reason of being unemployed, provided by the Place-based Longitudinal Data Resource (PLDR).^26 27^ This time-varying measure of small-area deprivation is the only measure of SECs at all that is available annually for neighbourhoods in England, and we treat it as a proxy for childhood SECs,^28^ in the absence of other suitable time-varying measures of SECs available at the neighbourhood level that would allow us to examine trends and relationships over time. See online supplemental figure S2 for biennial unemployment prevalence. Annual population estimates from the ONS were used to derive denominators for the outcome and exposure.^29^

To control for access to care, several indicators were provided by PLDR.^26^ We used time-varying measures of primary care access (the number of general practitioners (GPs) per capita serving the population (derived from NHS Digital data),^30^ and the proportion of the population who describe their experience of making a GP appointment as poor (derived from General Practice Patient Survey (GPPS) data)^31^; and secondary care access (the average road network distance to the nearest hospital with an ED).^32^ These have previously been used as proxy measures for access to care.^28^

Time-invariant baseline measures of ethnic composition, and the age-specific prevalence of long-term health problems per neighbourhood, derived from the 2011 census,^25^ were included in robustness tests (mixed-effects models) to account for potential confounding. There is evidence to show they are related to both deprivation and the hospitalisation outcomes.^8 14 33 34^

The logic model (online supplemental figure S1) describes the hypothetical causal pathway between income deprivation and emergency hospital admissions for paediatric ACSCs, and the potential effects of the covariates we had data for. Between-area differences in unmeasured variables that remain constant over the study period are accounted for automatically in fixed-effects models. A full description of the measures and data sources can be found in the supplementary file (online supplemental table S1).

### Statistical analyses

We used fixed-effects negative binomial regression models to investigate the association between annual changes in unemployment and changes in ACS emergency admissions between neighbourhoods, holding constant unobserved time-invariant characteristics for each neighbourhood. We fitted two models between 2012 and 2017 (restricted by data availability). Model 1 fixed negative binomial regression with unemployment and emergency admission rates (1) overall, (2) acute and (3) chronic ACSCs; stratified by age group adjusting for all unmeasured time-invariant confounding factors. Model 2 as per model 1 additionally adjusting for proxies of access to primary and secondary care.

The number of emergency admissions was modelled using the log of the population as an ‘offset’ variable, indicating the maximum number of admissions that could have occurred. Likelihood ratio tests supported the inclusion of a quadratic time trend. The models estimate the incident rate ratio (IRR), which is interpreted as the percentage change in the incident rate of emergency admissions associated with the changes in neighbourhood unemployment rate. The outcome was stratified by age group (0–9, 10–19 years) to account for different rates of hospital admissions related to ACSCs in children of different ages. Analyses were performed using R (V.4.3.0).

### Robustness tests

First, models 1 and 2 were repeated (2012–2017) using mixed-effects negative binomial regression. Mixed effects models included a random intercept to account for the longitudinal nature of the data (ie, observations clustering over time). These investigate the effect of unemployment changes while allowing for individual differences between neighbourhoods in baseline admission rates and are used as a robustness test to determine whether between-neighbourhood variation impacts the findings from the main analysis. This alternative method was used to assess the relationship between deprivation trends within neighbourhoods and trends in paediatric ACS emergency admission rates. Mixed effects models allow for direct adjustment of time-invariant confounders. We therefore stratified by age group adjusting for baseline ethnicity and proportion of population with chronic disease. Second, fixed effects model 1 (without mediators) was fitted between 2008 and 2019 to investigate the association between changes in neighbourhood unemployment and admission rates for ACSCs over a longer time period. We fitted our primary models over a shorter time frame from 2012 to 2017 because of the availability of mediator data over this period but were able to repeat our main fixed-effects analysis, without mediators, over a longer period as a sensitivity analysis. Third, to investigate the appropriateness of our chosen definition of ACSCs, we fitted models 1 and 2 using a narrower definition of ACSCs (using ICD-10 codes described in online supplemental figure S25) that was derived from existing literature.^35^

### Ethical considerations

No ethics approval was required for this study as it involved the use of anonymous aggregate secondary health service data and openly available data.

### Public involvement

The research focused on a quantitative analysis of existing routinely collected, anonymised data. Given the nature of this study, there were no direct interactions with patients or the public and patient involvement was not deemed necessary for the design, conduct or analysis of the research.

## Results

### Main analyses

**Figure 1:**
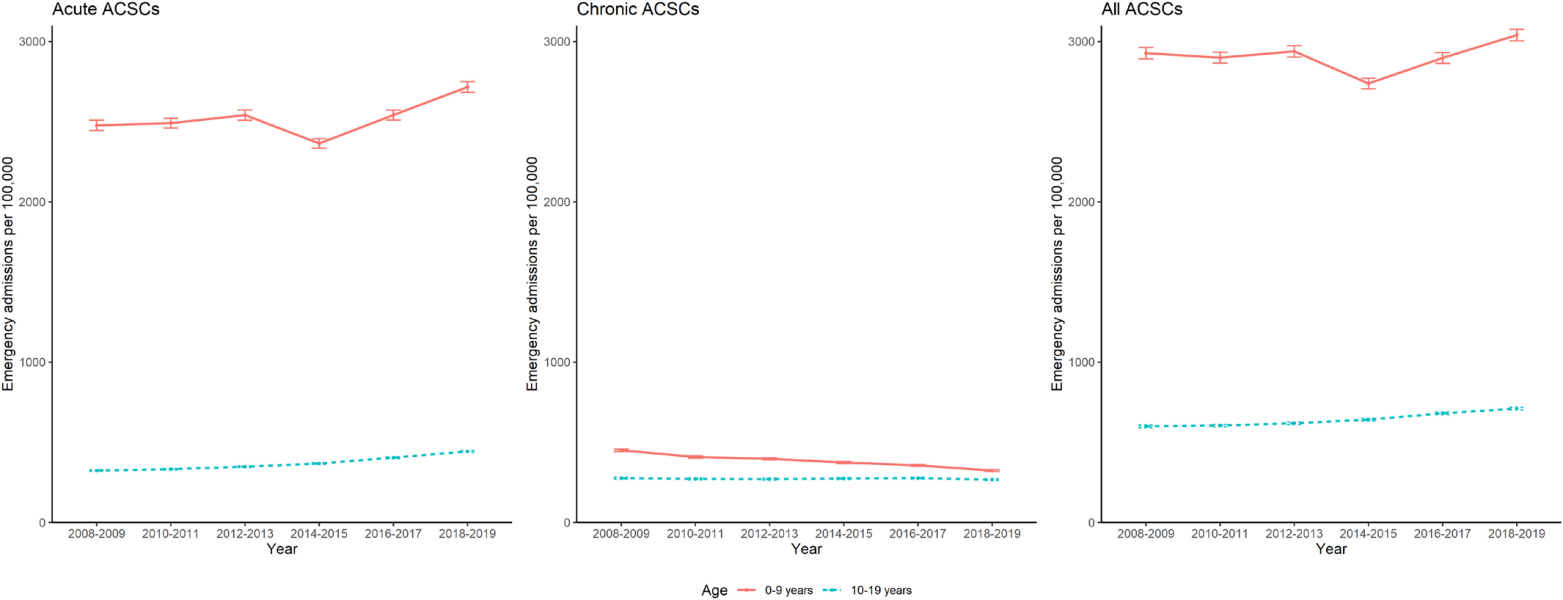
Emergency admission rates for English MSOAs (middle super output layers) for ambulatory care sensitive conditions (ACSCs) by age group, 2008–2019.

A total of 6771 (99.7%) English neighbourhoods had available outcome data from 2008 to 2019. Figure 1 shows emergency admission rates, per 100 000, for English neighbourhoods by age group for acute, chronic and all ACSCs, 2008–2019. There were considerably more admissions for acute ACSCs than chronic ACSCs. Younger children accounted for more admissions than older children. For acute ACSCs, average admission rates rose in younger children to 2736 per 100 000 between 2008 and 2019, an overall increase of 10%. For older children, rates increased by 37%, with an average admission rate of 444 per 100 000 in 2019. For chronic ACSCs, average admission rates decreased by 27% in younger children, and 3% in older children, over time. Unemployment prevalence decreased from 2.2% in 2012 to 1.4% in 2017 (see online supplemental figure S2 for biennial unemployment prevalence).

Complete data for all variables across all neighbourhoods were only available from 2012 to 2017, giving a total sample size of 20 313 MSOA-years for each age group. For children aged 0–9 years, there was a total of 996 322/150 701 emergency admissions of acute/chronic ACSCs in England between 2012 and 2017, respectively. The overall mean admissions across MSOAs and years was 49/7.4 for acute/chronic ACSCs. For children aged 10–19 years, there was a total of 139 457/102 289 acute/chronic ACSCs. The overall mean admissions across MSOAs and years was 6.9/5 for acute/chronic ACSCs. See supplementary file for biennial emergency admission rates (online supplemental tables S3–S5) and other characteristics of English neighbourhoods (online supplemental table S2).

Results from regression models show that increasing trends in neighbourhood unemployment were associated with increasing trends in ED admission rates for chronic, acute and all ACSCs. All models indicated this association was more pronounced for children aged under 10 years.

Table 1 presents results from fixed-effects regression models (not adjusted for mediators) for each outcome, by age group. For every additional 1% point increase in the population experiencing unemployment relating to low income, a 3.9% (IRR=1.039, 95% CI 1.032 to 1.046) and 2.7% (IRR=1.027, 95% CI 1.017 to 1.037) increase in admission rates for acute ACSCs would be expected for children aged 0–9 years and 10–19 years, respectively (approximately equivalent to an additional 12 856 and 1257 acute ACS admissions for children aged 0–9 years and 10–19 years, respectively, per 2 years for every 1% point increase in unemployment).

**Table 1:** Confounder-adjusted negative binomial model for emergency admissions by ACSC type and age group, 2012–2017

		Children aged 0–9 years			Sig	Children aged 10–19 years			Sig
		IRR	Lower 95% CI	Upper 95% CI		IRR	Lower 95% CI	Upper 95% CI	
ACS acute	Working age population unemployed (%)	1.039	1.032	1.046	^***^	1.027	1.017	1.037	^***^
ACS chronic	Working age population unemployed (%)	1.026	1.014	1.038	^***^	1.003	0.987	1.018	0.746
ACS all	Working age population unemployed (%)	1.038	1.031	1.044	^***^	1.017	1.008	1.027	^***^

Models include fixed effects for every two2 years.

Data based on 6771 MSOAs and 20 313 observations.

***p<0.01, **p<0.05, *p<0.1.

ACSCambulatory care sensitive conditionIRR, incident rate ratio; MSOA, middle layer super output area

For chronic ACSCs, this association had a magnitude of 2.6% (IRR=1.026, 95% CI 1.014 to 1.038) increase in admission rates, for children aged 0–9 years, respectively (approximately equivalent to an additional 1309 emergency admissions in 0–9 years old, every 2 years, per percentage point increase in unemployment). No significant association between unemployment and admissions for chronic ACSCs was observed for children aged 10–19 years (IRR=1.003, 95% CI 0.987 to 1.018).

For all ACSCs combined, this association had a magnitude of 3.8% (IRR=1.038, 95% CI 1.031 to 1.044) and 1.7% (IRR=1.017, 95% CI 1.008 to 1.027) increase in admission rates for children aged 0–9 years and 10–19 years, respectively (approximately 14 380 and 1405 additional emergency admissions for 0–9 years old and 10–19 years old, respectively, every 2 years).

Table 2 presents results from models additionally adjusted for primary and secondary care access. Across each outcome, the additional adjustment did not meaningfully attenuate the magnitude or statistical significance of the association between neighbourhood unemployment and ACS admission rates.

**Table 2:** Confounder-adjusted and mediator-adjusted negative binomial model for emergency admissions by ACSC type and age group, 2012–2017

		Children aged 0–9 years			Sig	Children aged 10–19 years			Sig
		IRR	Lower 95% CI	Upper 95% CI		IRR	Lower 95% CI	Upper 95% CI	
ACS acute	Working age population unemployed (%)	1.037	1.030	1.044	^***^	1.026	1.016	1.036	^***^
ACS chronic	Working age population unemployed (%)	1.027	1.015	1.039	^***^	1.003	0.987	1.019	0.733
ACS acute and chronic	Working age population unemployed (%)	1.036	1.030	1.043	^***^	1.017	1.008	1.026	^***^

Models include fixed effects for every two2 years.

Data based on 6771 MSOAs and 20 313 observations.

***p<0.01, **p<0.05, *p<0.1.

IRR, incident rate ratio; MSOA, middle layer super output area

### Sensitivity analyses

Results from mixed-effect models, were consistent with the primary analyses using mixed-effects models, in showing that increasing unemployment was associated with increasing admission rates. However, these models showed a larger effect magnitude in admission rates per percentage point increase in unemployment for all outcome measures (online supplemental tables S20 and S21).

Fixed-effects models estimated over a longer period (2008–2019) suggested the association between increased unemployment and increased admission rates for acute ACSCs and all ACSCs. However, over the extended time period, we no longer observed a significant association for children (0–9 years) with chronic ACSCs (online supplemental table S19). Results were also similar to the primary analysis, using a narrower definition for ACSCs (online supplemental tables S6, S22 and S23).

## Discussion

In this small-area ecological analysis of children in England between 2012 and 2017, increasing trends in neighbourhood unemployment were associated with increasing trends in emergency admission rates for all ACSCs in children. Adjusting for measures of primary and secondary care access did not have a significant effect on this association, supporting evidence that access to primary healthcare does not explain differences in avoidable ED use and therefore, policy interventions to improve primary care access may not be appropriate in decreasing avoidable emergency admissions.^36^

This study adds to the evidence base by showing increasing trends in emergency admissions for acute ACSCs in children, especially in younger children. Previous research found between 2001 and 2013, that while the rate of admissions for acute ACSCs increased, the rate for chronic ACSCs fell by 3%.^13^ This study provides similar findings; however, the decrease in emergency admissions for chronic ACSCs was much more dramatic for children. This study reflects findings that these increasing trends in ACSCs are primarily driven by acute ACSCs,^10^ and there is much variability in the trends seen when ACSCs are divided into two groups (acute and chronic). This further supports previous recommendations to analyse acute and chronic ACSCs separately.^13 14^

Stratifying by age showed this decline was more dramatic for younger children (a decrease of 27%), suggesting future work should further stratify by age. Younger children experienced more ACS emergency admissions than their older peers, supporting research suggesting an association between non-urgent ED visits and age.^14 37 38^ Children living in poverty are more likely to have a chronic illness.^39^ This study found, despite decreasing trends in admissions for chronic ACSCs, there was an association between neighbourhood unemployment and chronic ACSC emergency admissions for children aged 0–9 years. However, there was no observed association in chronic ACSCs for older children (10–19 years). This may be indicative of the differential impact of socioeconomic stressors (such as unemployment changes) on children of different ages. For example, younger children may have increased parental dependency and may be more vulnerable to unemployment changes that directly affect their caregivers. No association was observed for chronic ACSCs in both ages over a longer period, further suggesting that economic hardship may have a more immediate impact on acute health needs. For example, chronic ACSCs require more long-term care with the impact of these conditions manifesting over an extended period. This may create more long-standing relationships with health services, which could mitigate some immediate effects of changes in SECs.

We support previous findings of a social gradient in ACSCs ED admission,^13 19 22^ and showed that neighbourhood unemployment, used here as a proxy for childhood SECs,^28^ was a main predictor for increased avoidable emergency admissions. We provide evidence that increasing trends in unemployment are associated with increasing trends in emergency admissions for all ACSCs in children. This association was found despite an observed rise in admission and falling unemployment rates. However, this may be explained by the lagged effects of unemployment on health, for example, the compounding of the social determinants of health across vulnerable neighbourhoods. This study adds to the existing evidence base that suggests the principal lever to reduce these admissions is addressing underlying deprivation.

This is the first large-scale longitudinal analysis, using routinely available data and different methods to adjust for potential confounders and mediators, showing an association between increasing neighbourhood unemployment and emergency hospital admission rates for children in England with ACSCs. We build confidence in the causality between SEC and ACS hospitalisations by using the best available time-varying measure of SECs, measured at the MSOA level and therefore a reasonable proxy for individual SECs. While we cannot explicitly distinguish potentially avoidable admissions without individual case notes, our careful use of the defined ACSCs attempts to provide the best available indicator of ambulatory care.^40^

Multiple sensitivity analyses assessed the robustness of the main results. In attempting to mitigate the limitations of a comprehensive analysis within a small time period, robustness analyses across a wider time period of 2008–2019 remained suggestive that increasing neighbourhood unemployment was associated with increasing emergency admissions for acute and all ACSCs. However, extending the time period attenuated the observed association for children (0–9 years) with chronic ACSCs. The stronger effect of neighbourhood unemployment on ACS admissions between 2012 and 2017 could be potentially explained by the lag effect of austerity measures implemented by the government from 2010, with their full impact on communities becoming more evident over time. Mixed-effects models observed larger effect estimates. This could suggest that the confounder-adjusted mixed-effects model estimates are biased by unmeasured confounding. The use of fixed effects models adjusts more comprehensively for time-invariant confounding and therefore may give better causal estimates. Finally, using an alternative definition for ACSCs produced similar results to primary analyses, suggesting the definition was appropriate in identifying ACSCs. This also helped to mitigate the lack of an agreed definition for ACSCs.

Some limitations should be considered. Suppression due to small count numbers at MSOA level limited data to 2-year intervals, and broad age stratification. There is a possibility of ecological bias within this study, where associations found at the group level are not present at the individual level. These data do not account for repeat admissions, and we are unable to quantify how this may influence results. We use the only annual proxy measure of SECs; however, unemployment may not be the strongest indicator of neighbourhood SECs. While we attempt to capture access to primary and secondary care by using openly available proxy measures, there are also limitations to these measures and future research would benefit from investigating other access to health service data that may become available. MSOA level measures may introduce increased variation across these measures, which could be mitigated using smaller area estimates. While weighted GPPS data improves the representativeness, explicit demographic information on the responders to the surveys is unknown. A cautious approach towards conclusions of the mediating effect from these measures is therefore adopted. Further research using additional proxy measures (such as community-based care measures) would improve the interpretations drawn. Finally, this study does not use robust methods for exploring mediation, and so conclusions drawn on the mediating effect of variables used in this analysis are limited.^41^ A more robust approach for exploring mediation would be beneficial in future research to help mitigate barriers to inferring causality (such as a possible bidirectional relationship between SEC and the number of ACSC hospitalisations). This study indicates that paediatric ACS emergency hospitalisation disproportionately affects disadvantaged communities, especially for younger children, however, careful analysis of individual-level data from linked administrative databases would allow stronger conclusions to be drawn. Further studies should also examine other potential mediators. Qualitative and process evaluation studies should yield greater insight into pathways and solutions for mitigating the effects of disadvantageous SECs. Further research should investigate the mechanisms of socioeconomic disadvantage in more detail, to help to suggest effective policy interventions that aim to reduce avoidable emergency admissions.

Not only do ACS emergency admissions cause a financial strain on the NHS, but admission to the hospital is traumatic for both children and their families, where children admitted to the hospital are at risk of infections, medical errors, drug reactions and emotional trauma. Therefore, it is important to tackle this issue and reduce the burden of ACSCs.^42^ With evidence of rising child poverty, it is imperative to ensure equity of access to healthcare services for children, especially those who are disproportionately affected by adverse outcomes.

We show that increasing admissions in neighbourhood unemployment (as a proxy for SECs) were associated with increases in paediatric emergency admission rates for ACSCs, in England. With confirmation from robustness tests, we provide a comprehensive analysis of all paediatric ACS emergency hospital admissions in England over a 5-year period. Differential access to primary and secondary care did not appear to explain this relationship and therefore, policy interventions to improve primary care access may not be sufficient in decreasing ACS emergency admissions may not address socioeconomic inequalities.^36^ Reducing the inequality gap in emergency hospital admission rates for ACSCs should be a priority. Ultimately, the causes of unemployment and poverty need to be addressed to reduce the observed inequalities in health and healthcare suggested by this study.

## supplementary material

10.1136/bmjpo-2024-002991 online supplemental file 1

## Data Availability

Data are available in a public, open access repository. Data may be obtained from a third party and are not publicly available.
