# Supplementary material for: Neighbourhood socioeconomic conditions and emergency admissions for ambulatory care sensitive conditions in children: a longitudinal ecological analysis in England, 2012–2017
Source: BMJ Paediatr Open. 2025 Jan 19;9(1):e002991. doi: 10.1136/bmjpo-2024-002991 (PMC11749807; doi:10.1136/bmjpo-2024-002991)
Supplement: online supplemental file 1 [file bmjpo-9-1-s001.pdf]

Supplementary File

ACSC definition – Main analysis

Table S1. Ambulatory-care sensitive conditions and corresponding ICD-10 codes (Gill et al., 2013)

Condition	***ICD-10 code***
**Acute**	
Vaccine-preventable diseases	**A08.0***, A35-, A36-, A37-, A80-, B05-, B06-, B16.1, B16.9, B18.0, B18.1, B26-, G00.0, M01.4
Lower respiratory tract infections	J10.0, J11.0, J11.1, J12-, J13-, J14-, J15-, J16-, J18.0, J18.1, J18.9, J21-
Upper respiratory tract infections	H66-, H67-, J02-, J03-, J04.0, J06-, J31-
Dehydration and gastroenteritis	E86-, K52.2, K52.8, K52.9, A02.0, A04-, A07-, **A08-***, A09-
Urinary tract infections	N10-, N11-, N12-, N13.6, N15.9, N30.0, N30.8, N30.9, N39.0
**Chronic**	
Asthma	J45-, J46-
Diabetes	E10-, E11-, E12-, E13-, E14-
Epilepsy	G40-, G41-

Figure S1: Biennial unemployment prevalence – proportion of people aged 16–64 years claiming Jobseeker's Allowance or Universal Credit principally for the reason of being unemployed, for English neighbourhoods, 2008–19

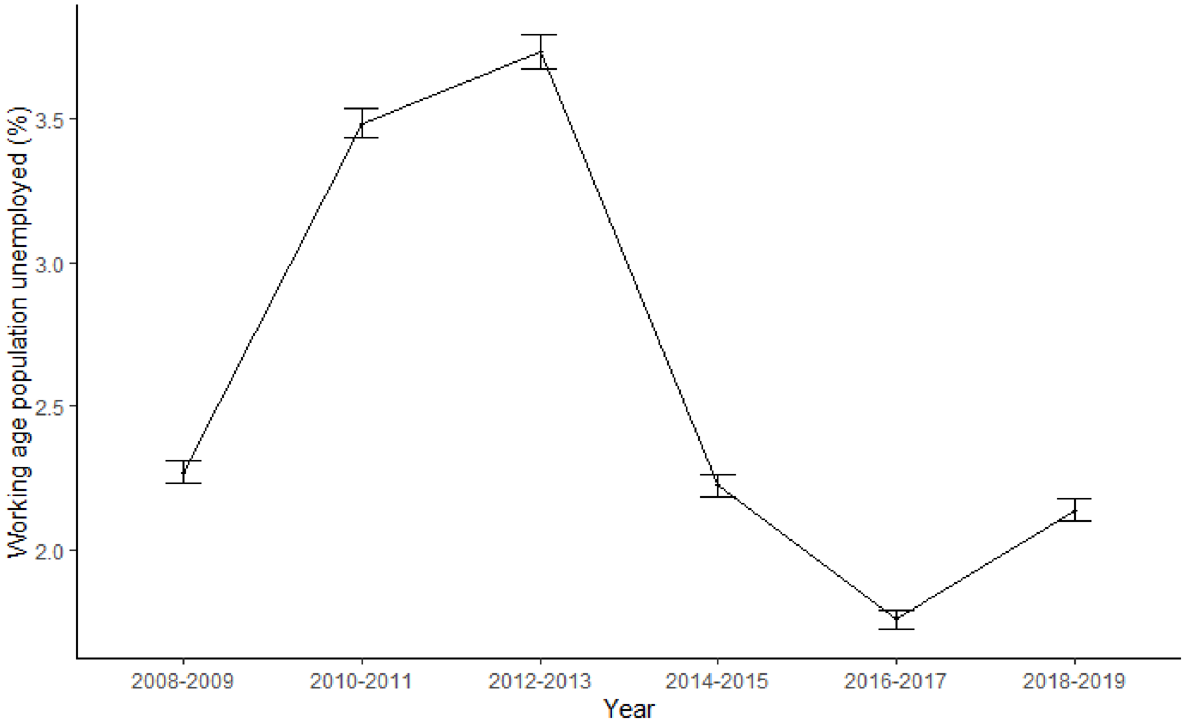

Figure S2: DAG

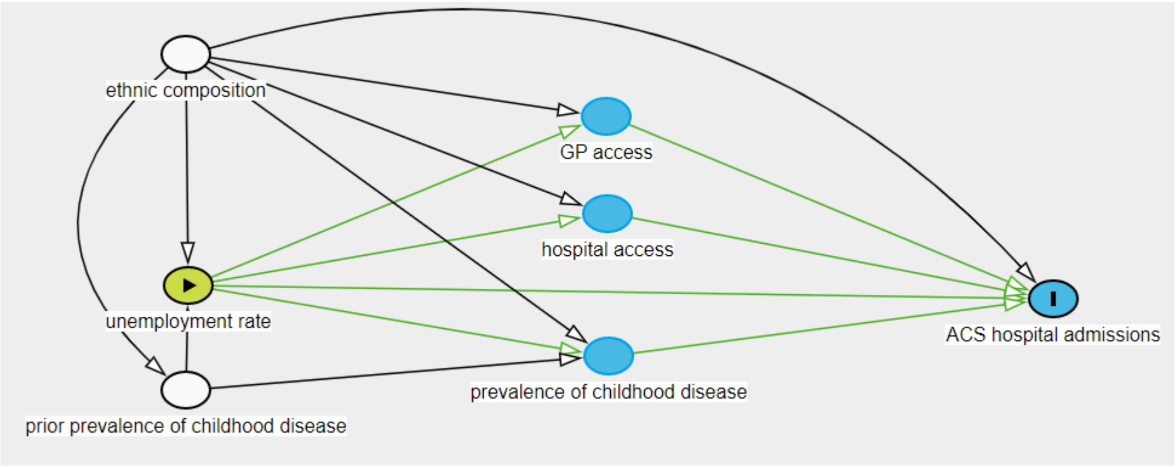

Table S2: Data sources and measures

The following variables were included in the analysis:

Variable	Description
Unemployment	Unemployment prevalence per year was calculated using claimant data provided by the ONS. Unemployment was measured as the percentage of people aged 16–64 years claiming Jobseeker's Allowance or Universal Credit principally for the reason of being unemployed. Biennial estimates from 2008-2018 were included in analysis.
ACS emergency hospital admissions	Emergency admissions for ACSCs were defined using ICD-10 codes. Total numbers of IID emergency admissions biennially for two age groups (0–9; 1-19 years) were calculated using Hospital Episode Statistics (HES). Data were provided by Place-based Longitudinal Data Resource (PLDR) under the data share agreement with NHS Digital (DARS-NIC-16656-D9B5T)
Prevalence long-term health problems (%)	The proportion of the population who report having a long-term health problem or disability was calculated for two age groups (0–9; 1-19 years) using census data. The 2011 Census defines a long-term health problem or disability as that which limits a person's day-to-day activities, and has lasted or is expected to last at least 12 months.
Population who would describe their experience of making a GP/nurse appointment as poor (%)	GP Patient Survey (GPPS) data were used to derive a measure for the proportion of the population who would describe their experience of making an appointment the last time they wanted to see or speak to a GP or nurse from their surgery, as fairly poor or very poor. These data were used to calculate weighted averages of the indicator by Lower Super Output Area (LSOA) and were available via the Place Based Longitudinal Data Resource (PLDR). Weighted averages of the indicator per MSOA and year were calculated using annual data provided by NHS Digital on the total number of patients registered per general practice per MSOA. Biennial estimates from 2013-2017 were included in analysis.
GPs per 1000 population	An indicator on GPs per capita was provided via the PLDR. Weighted averages of the

	number of full-time employed GPs per 1000 population were calculated using annual data provided by NHS Digital on the number of GPs and patients registered per general practice per LSOA. Biennial estimates from 2012-2019 were included in analysis.
Travelling distance to hospital with A&E (km)	An indicator on distance to hospital was provided via the PLDR per LSOA on the average road network distance to the nearest hospital with an Accident and Emergency (A&E) department. Road network distances in kilometres were calculated by deriving the fastest route by car to travel from each postcode within an LSOA to the nearest health service. This data was averaged per MSOA. Biennial estimates from 2011-2017 were included in analysis.
Ethnic group (%)	The proportion of the population who classify their own perceived ethnic group and cultural background as: White / White British / White Irish / White gypsy / White other; Asian British / Asian British Indian/ Asian British Pakistani/ Asian British Bangladeshi / Asian British Chinese/ Asian British other; Black / African / Caribbean / Black British / any other Black / African / Caribbean background; Mixed / White and Black Caribbean / White and Black African / White and Asian / any other mixed ethnic group ;Other/ Arab/ any other ethnic group. The ONS provide data about the ethnic group of the usual resident population of England as at census day, 27 March 2011.

ACSC definition – Sensitivity analysis

Table S3: Ambulatory care sensitive admission ICD-10 codes (Cecil et al., 2018)

Description	ICD-10 codes
**Chronic conditions**	
Asthma	J45, J46
Diabetes	E10-E14
Epilepsy	G40, G41
**Acute infections**	

Lower respiratory tract infections	J10.0, J11.0, J11.1, J12-J16, J18.0, J18.1, J18.9, J21
Upper respiratory tract infections	H66, H67, J02, J03, J04.0, J06, J31.2
Dehydration and gastroenteritis	E86, K52.2, K52.8, K52.9, A02.0, A04, A07.2, A08.0, A08.1, A08.3, A08.4, A08.5, A09
Urinary tract infections	N10-N12, N13.6, N15.9, N30.0, N30.8, N30.9, N39.0
**Other**	
Vaccine-preventable diseases	A35-A37, A80, B05, B06, B16.1, B16.9, B18.0, B18.1, B26, G00.0, M01.4

Table S4: Biennial emergency admissions for chronic ACS conditions per 100,000 population

	Children aged 0-9 years			Children aged 10-19 years		
Year	Mean	Lower 95% CI	Upper 95% CI	Mean	Lower 95% CI	Upper 95% CI
2008-2009	440	433	447	276	271	282
2010-2011	397	390	404	273	267	278
2012-2013	388	382	395	272	267	277
2014-2015	366	360	373	274	268	279
2016-2017	353	346	358	276	271	282
2018-2019	318	313	324	268	263	273

Data based on 6,771 MSOAs. Values rounded to the nearest whole number.
CI = confidence interval; MSOA = Middle layer super output area

Table S5: Biennial emergency admissions for acute ACS conditions per 100,000 population

Year	Children aged 0-9 years			Children aged 10-19 years		
	Mean	Lower 95% CI	Upper 95% CI	Mean	Lower 95% CI	Upper 95% CI
2008-2009	2477	2445	2509	325	320	329
2010-2011	2493	2463	2523	334	330	338
2012-2013	2544	2512	2576	349	345	354
2014-2015	2369	2339	2398	369	364	374
2016-2017	2562	2531	2593	407	402	412
2018-2019	2736	2702	2769	444	439	450

Data based on 6,771 MSOAs. Values rounded to the nearest whole number.
CI = confidence interval; MSOA = Middle layer super output area

Table S6: Biennial emergency admissions for all ACS conditions per 100,000 population

Year	Children aged 0-9 years			Children aged 10-19 years		
	Mean	Lower 95% CI	Upper 95% CI	Mean	Lower 95% CI	Upper 95% CI
2008-2009	2917	2882	2952	601	593	609
2010-2011	2890	2857	2923	607	599	614
2012-2013	2932	2897	2967	621	614	629
2014-2015	2735	2703	2767	642	634	650
2016-2017	2914	2880	2947	684	675	692
2018-2019	3054	3018	3090	712	704	720

Data based on 6,771 MSOAs. Values rounded to the nearest whole number.
CI = confidence interval; MSOA = Middle layer super output area

Table S7: Characteristics of English MSOAs between 2012-2017

	Mean (SD)
Working age population unemployed (%)	2.52 (2.08)
Prevalence long-term health problems children aged 0-9 years (%)	2.94 (0.998)
Prevalence long-term health problems children aged 10-19 years (%)	4.88 (1.47)
Population who would describe their experience of making a GP/nurse appointment as poor (%)	11.65 (5.63)
GPs per 1000 population	0.52 (0.096)
Travelling distance to hospital with A&E (km)	6.62 (5.35)
Ethnic group: Asian (%)	7.20 (11.95)
Ethnic group: Black (%)	3.26 (6.33)
Ethnic group: White (%)	86.39 (17.93)
Ethnic group: Mixed (%)	2.18 (1.77)
Ethnic group: Other (%)	0.97 (1.64)

Data based on 6,771 MSOAs
A&E = Accident and Emergency department; GP = general practitioner; km = kilometres; SD = standard deviation; MSOA = Middle layer super output area

Robustness tests – mixed-effects models

Table S8: Confounder adjusted mixed-effect negative binomial regression model showing the effect of change in unemployment on change in emergency admissions for chronic ACS conditions, acute ACS conditions, and all ACS conditions for English MSOAs, 2012–2017

	Children aged 0-9 years			Children aged 10-19 years		
	IRR	Lower 95% CI	Upper 95% CI	IRR	Lower 95% CI	Upper 95% CI
Acute ACSCs	1.067	1.062	1.072	1.057	1.052	1.062
Chronic ACSCs	1.059	1.052	1.066	1.052	1.044	1.060
All ACSCs	1.064	1.060	1.069	1.054	1.049	1.060

Models include random intercept for MSOA,
Data based on 6,771 MSOAs and 20,313 observations.
CI = confidence interval; IRR = incident rate ratio; MSOA = Middle layer super output area

Table S9: Confounder and mediator adjusted mixed-effect negative binomial regression model showing the effect of change in unemployment on change in emergency admissions for chronic ACS conditions, acute ACS conditions, and all ACS conditions for English MSOAs, 2012–2017

	Children aged 0-9 years			Children aged 10-19 years		
	IRR	Lower 95% CI	Upper 95% CI	IRR	Lower 95% CI	Upper 95% CI
Acute ACSCs	1.066	1.061	1.071	1.054	1.049	1.060
Chronic ACSCs	1.058	1.051	1.065	1.049	1.041	1.057
All ACSCs	1.064	1.059	1.068	1.052	1.047	1.057

Models include random intercept for MSOA,
Data based on 6,771 MSOAs and 20,313 observations.
CI = confidence interval; IRR = incident rate ratio; MSOA = Middle layer super output area

Robustness tests – longer time period 2008-2019

Table S10: Fixed-effect negative binomial regression model showing the effect of change in unemployment on change in emergency admissions for chronic ACS conditions, acute ACS conditions, and all ACS conditions for English MSOAs, 2008–2019

	Children aged 0-9 years			Children aged 10-19 years		
	IRR	Lower 95% CI	Upper 95% CI	IRR	Lower 95% CI	Upper 95% CI
Acute ACS conditions	1.041	1.037	1.045	1.014	1.009	1.019
Chronic ACS conditions	1.005	0.999	1.012	0.996	0.989	1.004
All ACS conditions	1.037	1.033	1.040	1.008	1.004	1.013

Models include fixed effects for every two years.
Data based on 6,771 MSOAs and 40,626 observations.
CI = confidence interval; IRR = incident rate ratio; MSOA = Middle layer super output area

Robustness tests – Using a different definition

Table S11: Biennial emergency admissions for ACS conditions (sensitivity definition) per 100,000 population

	Children aged 0-9 years			Children aged 10-19 years		
Year	Mean	Lower 95% CI	Upper 95% CI	Mean	Lower 95% CI	Upper 95% CI
2008-2009	2914	2879	2949	599	591	606
2010-2011	2886	2852	2919	604	596	611
2012-2013	2928	2893	2963	617	610	625
2014-2015	2731	2699	2764	640	632	648
2016-2017	2911	2877	2944	682	673	690
2018-2019	3049	3013	3084	710	702	718

Data based on 6,771 MSOAs. Values rounded to the nearest whole number.
CI = confidence interval; MSOA = Middle layer super output area

Table S12: Fixed-effect negative binomial regression model showing the effect of change in unemployment on change in emergency admissions for all ACS conditions for English MSOAs, 2012–2017

	Children aged 0-9 years			Children aged 10-19 years		
	IRR	Lower 95% CI	Upper 95% CI	IRR	Lower 95% CI	Upper 95% CI
Working age population unemployed (%)	1.038	1.031	1.044	1.018	1.009	1.028

Models include fixed effects for every two years.
Data based on 6,771 MSOAs and 20,313 observations.
CI = confidence interval; IRR = incident rate ratio; MSOA = Middle layer super output area

Table S13: Mediator adjusted fixed-effect negative binomial regression model showing the effect of change in unemployment on change in emergency admissions for all ACS conditions for English MSOAs, 2012–2017

	Children aged 0-9 years			Children aged 10-19 years		
	IRR	Lower 95% CI	Upper 95% CI	IRR	Lower 95% CI	Upper 95% CI
Working age population unemployed (%)	1.036	1.030	1.043	1.018	1.009	1.027

Models include fixed effects for every two years.

Data based on 6,771 MSOAs and 20,313 observations.

CI = confidence interval; IRR = incident rate ratio; MSOA = Middle layer super output area

Figure S3: Emergency hospital admission rates for ACSCs (sensitivity definition) by age group, 2008-19

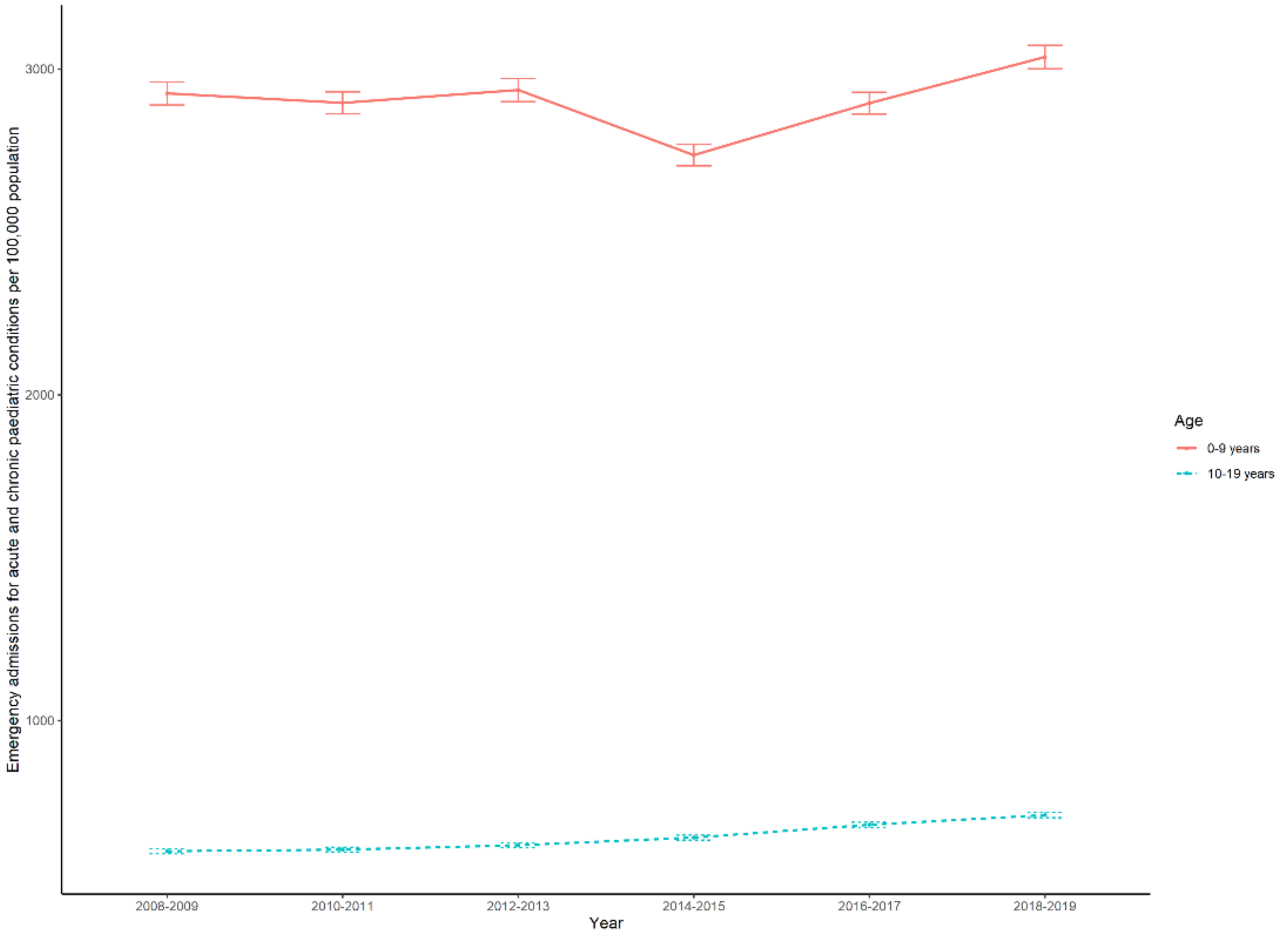

Figure S4: Correlation matrix of covariates

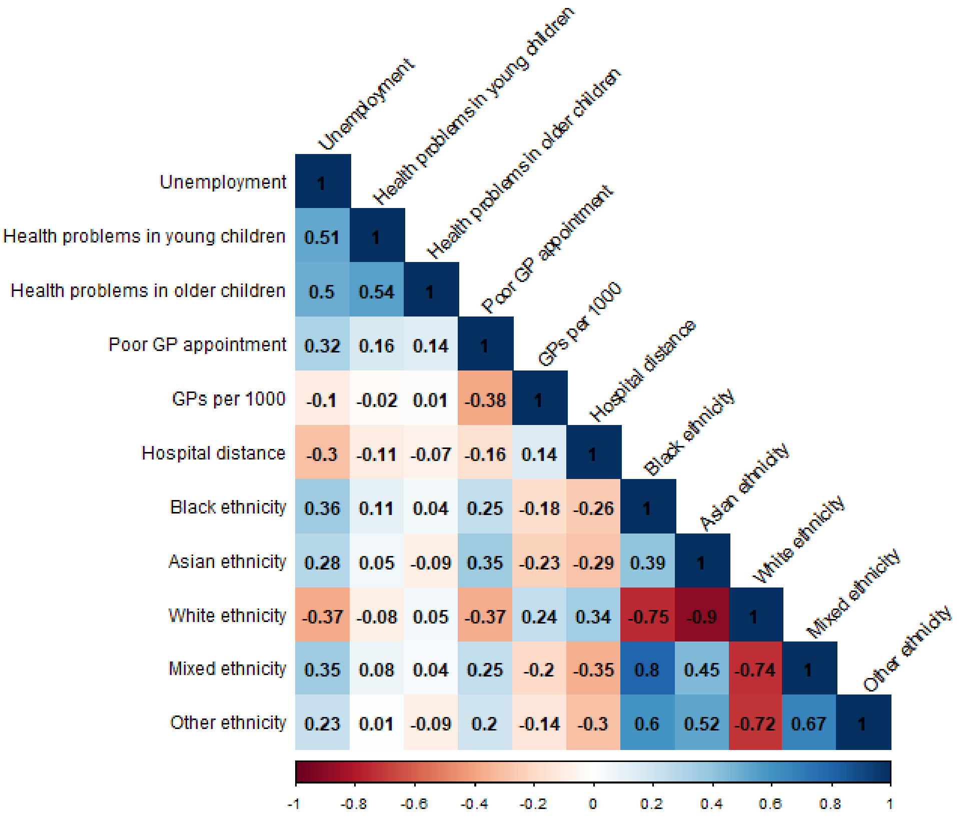

Models – main analysis

Age group: 0-9 years

Table S14: Fixed-effect negative binomial regression model showing the effect of change in unemployment on change in emergency admissions for acute ACS conditions children aged 0–9 years, for English MSOAs, 2012–2017

	Estimate	St.Err	z-value	p-value	Sig
Working age population unemployed (%)	0.037979	0.003480	10.9133	< 2.2e-16	***
Year	-0.414310	0.031138	-13.3057	< 2.2e-16	***
Quadratic year term	0.057192	0.003590	15.9313	< 2.2e-16	***

Models include fixed effects for every two years.
Data based on 6,771 MSOAs and 20,313 observations.
CI = confidence interval; IRR = incident rate ratio; MSOA = Middle layer super output area
Adjusted pseudo R^2 = 0.17, BIC = 212,433.2
*** p<0.01, ** p<0.05, * p<0.1

Table S15: Mediator adjusted fixed-effect negative binomial regression model for emergency admissions for acute ACS conditions children aged 0–9 years, for English MSOAs, 2012–2017

	Estimate	St.Err	z-value	p-value	Sig
Working age population unemployed (%)	0.036499	0.003480	10.48690	< 2.2e-16	***
Population who would describe their experience of making a GP/nurse appointment as poor (%)	-0.002600	0.000761	-3.41550	0.00063664	***
GPs per 1000 population	0.055282	0.037871	1.45975	0.14435845	
Travelling distance to hospital with A&E (km)	-0.002117	0.000856	-2.47438	0.01334690	*
Year	-0.415437	0.032909	-12.62385	< 2.2e-16	***
Quadratic year term	0.057648	0.003832	15.04420	< 2.2e-16	***

Models include fixed effects for every two years.
Data based on 6,771 MSOAs and 20,313 observations.
CI = confidence interval; IRR = incident rate ratio; MSOA = Middle layer super output area
Adjusted pseudo R^2 = 0.17, BIC = 212,431.4
*** p<0.01, ** p<0.05, * p<0.1

Table S16: Fixed-effect negative binomial regression model showing the effect of change in unemployment on change in emergency admissions for chronic ACS conditions children aged 0–9 years, for English MSOAs, 2012–2017

	Estimate	St.Err	z-value	p-value	Sig
Working age population unemployed (%)	0.025895	0.005906	4.384425	1.1629e-05	***
Year	0.014768	0.066291	0.222778	8.2371e-01	
Quadratic year term	-0.004649	0.007843	-0.59273	5.5336e-01	

Models include fixed effects for every two years.
Data based on 6,771 MSOAs and 20,313 observations.
CI = confidence interval; IRR = incident rate ratio; MSOA = Middle layer super output area
Adjusted pseudo R^2 = 0.10, BIC = 162,520.4
*** p<0.01, ** p<0.05, * p<0.1

Table S17: Mediator adjusted fixed-effect negative binomial regression model for emergency admissions for chronic ACS conditions children aged 0–9 years, for English MSOAs, 2012–2017

	Estimate	St.Err	z-value	p-value	Sig
Working age population unemployed (%)	0.026263	0.005943	4.419016	9.9151e-06	***
Population who would describe their experience of making a GP/nurse appointment as poor (%)	-0.000064	0.001529	-0.041741	9.6671e-01	
GPs per 1000 population	-0.057086	0.073060	-0.781354	4.3459e-01	
Travelling distance to hospital with A&E (km)	-0.003082	0.002145	-1.437179	1.5067e-01	
Year	0.027650	0.070181	0.393976	6.9360e-01	
Quadratic year term	-0.006308	0.008366	-0.753964	4.5087e-01	

Models include fixed effects for every two years.
Data based on 6,771 MSOAs and 20,313 observations.
CI = confidence interval; IRR = incident rate ratio; MSOA = Middle layer super output area
Adjusted pseudo R^2 = 0.10, BIC = 162,545.7
*** p<0.01, ** p<0.05, * p<0.1

Table S18: Fixed-effect negative binomial regression model showing the effect of change in unemployment on change in emergency admissions for all ACS conditions children aged 0–9 years, for English MSOAs, 2012–2017

	Estimate	St.Err	z-value	p-value	Sig
Working age population unemployed (%)	0.036917	0.003219	11.4697	< 2.2e-16	***
Year	-0.355291	0.028981	-12.2595	< 2.2e-16	***
Quadratic year term	0.048835	0.003345	14.5986	< 2.2e-16	***

Models include fixed effects for every two years.
Data based on 6,771 MSOAs and 20,313 observations.
CI = confidence interval; IRR = incident rate ratio; MSOA = Middle layer super output area
Adjusted pseudo R^2 = 0.17, BIC = 215,791.3
*** p<0.01, ** p<0.05, * p<0.1

Table S19: Mediator adjusted fixed-effect negative binomial regression model for emergency admissions for all ACS conditions children aged 0–9 years, for English MSOAs, 2012–2017

	Estimate	St.Err	z-value	p-value	Sig
Working age population unemployed (%)	0.035702	0.003222	11.07983	< 2.2e-16	***
Population who would describe their experience of making a GP/nurse appointment as poor (%)	-0.002257	0.000718	-3.14493	0.0016612	***
GPs per 1000 population	0.040166	0.035201	1.14105	0.2538492	
Travelling distance to hospital with A&E (km)	-0.002153	0.000834	-2.58137	0.0098409	***
Year	-0.354374	0.030724	-11.5339	< 2.2e-16	***
Quadratic year term	0.048989	0.003582	13.67545	< 2.2e-16	***

Models include fixed effects for every two years.
Data based on 6,771 MSOAs and 20,313 observations.
CI = confidence interval; IRR = incident rate ratio; MSOA = Middle layer super output area
Adjusted pseudo R^2 = 0.17, BIC = 215,792.7
*** p<0.01, ** p<0.05, * p<0.1

Age group: 10-19 years

Table S20: Fixed-effect negative binomial regression model showing the effect of change in unemployment on change in emergency admissions for acute ACS conditions children aged 10–19 years, for English MSOAs, 2012–2017

	Estimate	St.Err	z-value	p-value	Sig
Working age population unemployed (%)	0.026682	0.005023	5.311746	1.0858e-07	***
Year	0.068454	0.057321	1.194217	2.3239e-01	
Quadratic year term	0.004625	0.006828	0.677326	4.9820e-01	

Models include fixed effects for every two years.
Data based on 6,771 MSOAs and 20,313 observations.
CI = confidence interval; IRR = incident rate ratio; MSOA = Middle layer super output area
Adjusted pseudo R^2 = 0.07, BIC = 157,351.1
*** p<0.01, ** p<0.05, * p<0.1

Table S21: Mediator adjusted fixed-effect negative binomial regression model for emergency admissions for acute ACS conditions children aged 10–19 years, for English MSOAs, 2012–2017

	Estimate	St.Err	z-value	p-value	Sig
Working age population unemployed (%)	0.025588	0.005049	5.068255	4.0148e-07	***
Population who would describe their experience of making a GP/nurse appointment as poor (%)	-0.000874	0.001239	-0.705138	4.8072e-01	
GPs per 1000 population	0.078523	0.063729	1.232127	2.1790e-01	
Travelling distance to hospital with A&E (km)	0.003640	0.001646	2.210793	2.7050e-02	**
Year	0.055393	0.060620	0.913762	3.6084e-01	
Quadratic year term	0.006377	0.007260	0.878304	3.7978e-01	

Models include fixed effects for every two years.
Data based on 6,771 MSOAs and 20,313 observations.
CI = confidence interval; IRR = incident rate ratio; MSOA = Middle layer super output area
Adjusted pseudo R^2 = 0.07, BIC = 157,371.8
*** p<0.01, ** p<0.05, * p<0.1

Table S22: Fixed-effect negative binomial regression model showing the effect of change in unemployment on change in emergency admissions for chronic ACS conditions children aged 10–19 years, for English MSOAs, 2012–2017

	Estimate	St.Err	z-value	p-value	Sig
Working age population unemployed (%)	0.002584	0.007991	0.323356	0.74643	
Year	-0.008120	0.081997	-0.099028	0.92112	
Quadratic year term	0.002549	0.009695	0.262878	0.79264	

Models include fixed effects for every two years.
Data based on 6,771 MSOAs and 20,313 observations.
CI = confidence interval; IRR = incident rate ratio; MSOA = Middle layer super output area
Adjusted pseudo R^2 = 0.06, BIC = 154,039.3
*** p<0.01, ** p<0.05, * p<0.1

Table S23: Mediator adjusted fixed-effect negative binomial regression model for emergency admissions for chronic ACS conditions children aged 10–19 years, for English MSOAs, 2012–2017

	Estimate	St.Err	z-value	p-value	Sig
Working age population unemployed (%)	0.002736	0.008017	0.341218	0.73294	
Population who would describe their experience of making a GP/nurse appointment as poor (%)	-0.002043	0.001869	-1.093550	0.27415	
GPs per 1000 population	-0.156085	0.095478	-1.634781	0.10210	
Travelling distance to hospital with A&E (km)	0.000722	0.002505	0.288406	0.77304	
Year	0.045731	0.085934	0.532164	0.59461	
Quadratic year term	-0.004188	0.010226	-0.409538	0.68215	

Models include fixed effects for every two years.
Data based on 6,771 MSOAs and 20,313 observations.
CI = confidence interval; IRR = incident rate ratio; MSOA = Middle layer super output area
Adjusted pseudo R^2 = 0.06, BIC = 154,063.2
*** p<0.01, ** p<0.05, * p<0.1

Table S24: Fixed-effect negative binomial regression model showing the effect of change in unemployment on change in emergency admissions for all ACS conditions children aged 10–19 years, for English MSOAs, 2012–2017

	Estimate	St.Err	z-value	p-value	Sig
Working age population unemployed (%)	0.017280	0.004577	3.775616	0.00015961	***
Year	0.048000	0.048884	0.981919	0.32613965	
Quadratic year term	0.002416	0.005792	0.417192	0.67653804	

Models include fixed effects for every two years.
Data based on 6,771 MSOAs and 20,313 observations.
CI = confidence interval; IRR = incident rate ratio; MSOA = Middle layer super output area
Adjusted pseudo R^2 = 0.09, BIC = 173,031.0
*** p<0.01, ** p<0.05, * p<0.1

Table S25: Mediator adjusted fixed-effect negative binomial regression model for emergency admissions for all ACS conditions children aged 10–19 years, for English MSOAs, 2012–2017

	Estimate	St.Err	z-value	p-value	Sig
Working age population unemployed (%)	0.016773	0.004592	3.652613	0.00025959	***
Population who would describe their experience of making a GP/nurse appointment as poor (%)	-0.001419	0.001098	-1.292290	0.19625683	
GPs per 1000 population	-0.026807	0.055966	-0.478986	0.63194856	
Travelling distance to hospital with A&E (km)	0.002528	0.001577	1.603121	0.10890785	
Year	0.065163	0.051129	1.274471	0.20249642	
Quadratic year term	0.000339	0.006090	0.055731	0.95555585	

Models include fixed effects for every two years.
Data based on 6,771 MSOAs and 20,313 observations.
CI = confidence interval; IRR = incident rate ratio; MSOA = Middle layer super output area
Adjusted pseudo R^2 = 0.09, BIC = 173,053.5
*** p<0.01, ** p<0.05, * p<0.1
