# Supplementary material for: Neighbourhood socioeconomic conditions and emergency admissions for ambulatory care sensitive conditions in children: a longitudinal ecological analysis in England, 2012–2017
Source: BMJ Paediatr Open. 2025 Jan 19;9(1):e002991. doi: 10.1136/bmjpo-2024-002991 (PMC11749807; doi:10.1136/bmjpo-2024-002991)
Supplement: Supplementary file 2 [file bmjpo-2024-002991.reviewer_comments.pdf]

PEER REVIEW HISTORY

BMJ Paediatrics Open publishes all reviews undertaken for accepted manuscripts. Reviewers are asked to complete a checklist review form and are provided with free text boxes to elaborate on their assessment. These free text comments are reproduced below.

ARTICLE DETAILS

**TITLE (PROVISIONAL)**	Neighbourhood socioeconomic conditions and emergency admissions for ambulatory care sensitive conditions in children. A longitudinal ecological analysis in England, 2012-2017
**AUTHORS**	franklin, courtney; Mason, Kate; Akanni, Lateef; Daras, Konstantinos; Rose, Tanith; Carter, Bernie; Carrol, Enitan; Taylor-Robinson, David

VERSION 1 - REVIEW

**REVIEWER NAME**	***Ingrid Wolfe***
**REVIEWER AFFILIATION**	King's College London, Women's and Children's Health
**REVIEWER CONFLICT OF INTEREST**	
**DATE REVIEW RETURNED**	24-Sep-2024

**GENERAL COMMENTS**	We'd first like to thank you for the opportunity to review this paper. It is a cleverly constructed study with the particularly nice feature of capturing a temporal measure of socioeconomic conditions. The paper itself is clear and well-written, and we're glad to see this work being done. We think this submission is suitable for BMJ Paediatrics.<br><br>We've made suggestions for revisions or clarifications, section by section.<br><br>Abstract:<br>• Pulling sentence one from the introduction into the abstract could aid readers less familiar with ACSCs.<br>• The conclusion states the main effect was not explained by differential access to care - this is perhaps a little too strongly worded given how hard it is to measure access to care, and due to the strength of the measure for secondary care access used.<br>Intro:<br>• Suggest a clarifying statement about the fact that ACSCs, avoidable admissions, preventable hospitalisations, avoidable hospitalisations have been used as interchangeable concepts in the literature to mean hospitalisations which could have been avoided with timely effective outpatient (not necessarily primary) care. The distinction is important as, although it's hard to pin down an agreed definition, for children, diabetes and epilepsy are rarely managed in primary care so the one currently presented may be confusing to readers.<br>• Suggest clarifying note that some are unavoidable e.g. severe asthma exacerbations<br><br>Methods:<br>• Clarification requested as to whether this exposure is the only area-level time-varying proxy for SEC at all in the UK, or if it is the

	only one publicly available, the only one from the sources available to the authors, etc.<br>• The measures for access to primary care are quite nice in combination. Concerns that at MSOA level there may be a lot of variation, and also as to who responds to the GPPS surveys. If they are known to be representative, a line to give that reassurance would be really nice. The secondary care measure is much weaker, however, we appreciate this is hard to capture and it is still a nice addition as a secondary analysis, as long as the conclusions drawn are not too strong.<br>• It does not seem that access to care is necessarily strictly a mediator, and as it has only been controlled for, not subject to mediation analysis (as far as we can see in the paper), some additional justification here could be useful. It seems plausible that poor access to care leads to higher unemployment as well as more ACSC admissions, and that some access issues are less problematic if you are not currently employed, etc.<br>• The text states "we additionally adjusted for access to care measures, to evaluate whether these were likely to be mediating". We think this is a little unclear as if we are assessing whether they are mediators, a mediation analysis would be better. If we're simply trying to isolate the main effect, then this analysis is OK. Further clarity on the methods used may be sufficient to address all the comments on mediation.<br><br>Results:<br>• Tables are to a different degree of precision to the text (3.dp vs 2.dp).<br>• P-values and standard * approach available in supplementary tables, but not in the main manuscript. Is there a reason for not including the *? CIs helpful, but nice to get a simpler indication that results are comfortably significant without having to check the supplementary materials.<br><br>Discussion:<br>• The line that states "we overcome the challenges faced by earlier studies to inferring causality…" is too strong. This is a much nicer design to build confidence in the causality, but we do not believe it is fair to say the challenges have been overcome. There are still barriers to inferring causality e.g., strength of the proxy for SEC, a risk of endogeneity through unmeasured confounders/mediators and a potential for a bidirectional relationship between SEC and the number of ACSC hospitalisations, etc.<br>• Though the statement is accurate and fair, we think including 'Conservative-led' risks politicising the paper in a way that could detract from the impartiality of the important conclusions it draws.<br>• 'may give better causal estimates' would be preferable to 'may give the best causal estimates', even if within the context of this study, they may be the best.<br>• The null result (ACS Chronic, 10-19Yr olds) is restated but not explored in any detail the discussion. Not a necessity, but some discussion of this would be a nice addition.<br>• Typo – 'Theses data do not account for…'<br><br>Reviewers: Ingrid Wolfe (Professor of Paediatrics and Child Health), Lucy Pickard (Consultant Paediatrician, PhD student), Dylan Clarke (PhD student)

**REVIEWER NAME**	***Michelle Heys***
**REVIEWER AFFILIATION**	University College London Institute of Child Health
**REVIEWER CONFLICT OF INTEREST**	
**DATE REVIEW RETURNED**	17-Oct-2024

**GENERAL COMMENTS**	Summary<br><br>This ecological study examines the relationship between neighbourhood deprivation, measured by unemployment rates, and hospital/emergency admission rates for ambulatory care sensitive conditions (ACSC) in children aged 0-19 years over a five-year period (2012-2017) in England. Understanding the link between poverty, health, and health service use is crucial, and this analysis aims to quantify elements of these relationships.<br><br>Key findings include<br><br>Emergency admission rates increased over time, particularly for acute ACSC (reported over 2008-2019). However, the main analysis period (2012-2017) shows little variation in acute ACSC admission rates and an increase in chronic ACSC.<br><br>Neighbourhood unemployment rates decreased from approximately 3.5% to 1% during 2012-2017, though this is not detailed in the results.<br><br>Emergency admission rates were associated with unemployment rates for all groups except children aged 10-19 with chronic ACSC. A 1% change in unemployment rates corresponded to a ~3% increase in ACSC admission rates, equivalent to a doubling of unemployment rates by the end of the period.<br><br>Adjustments for primary and secondary care access proxies did not alter these associations.<br><br>In the main regression analyses, unemployment rates were positively associated with admission rates, while the time period was negatively associated. Adjustments for baseline ethnicity and prevalence of long-term conditions did not impact these associations, nor did proxies for access to care. These associations were most evident in the 0-9 years age group. Sensitivity analyses had minimal impact on the associations.<br><br>The study makes a valuable contribution to understanding the impact of socio-economic status on hospital use. However, significant revisions are needed, particularly in the methods and results sections, to better describe the work.<br><br>Strengths and Importance<br><br>The study addresses a significant public health issue by exploring the impact of socio-economic factors on health outcomes in children. The use of multiple datasets (HES, ONS, PLDR, GPPS, and the 2011 Census) across most of England provides a comprehensive approach to understanding these relationships. Delineating acute v chronic conditions is also a helpful approach to potentially understanding drivers of potentially avoidable health care use.

	Areas for Improvement<br><br>While the topic is important, the methods and results sections could benefit from clearer presentation to enhance understanding.<br><br>Abstract<br><br>The abstract effectively highlights the study's focus but could be more balanced in discussing the impact of emergency admissions. For instance, while ACS emergency admissions can strain the NHS and be traumatic for children, they can also resolve underlying health issues.<br><br>The conclusion in the abstract might overstate the findings. A more accurate reflection could be: "This was not explained by available measures of differential access to care, suggesting policy interventions should address the causes of unemployment and poverty in addition to health system factors to reduce emergency admissions for ACSCs."<br><br>Introduction<br><br>It would be helpful to describe the proportion of emergency admissions that are ACSC versus non-ACSC to provide context.<br><br>Methods<br><br>No additional analysis recommended, merely tighter clearer description and reporting of analysis.<br><br>The methods section needs more clarity. It is not fully clear whether the authors are examining ED attendance or hospital admissions. Clarifying this distinction is crucial.<br><br>A narrative around the DAG should be included in the main text.<br><br>A detailed description of the datasets and the conditions included in the acute and chronic categories should be provided in the main text.<br><br>The primary outcome of interest should be clearly stated, and the rationale for using biennial admission rates instead of annual rates should be explained.<br><br>More information on the data and measures used would be beneficial. For example, a simple statement outlining the datasets and their specific uses could improve clarity.<br><br>Regression models need to be more clearly described and outcome clarified (is the main outcome the delta variation in admission rates and proportion?) e.g. main analysis: "Model 1 fixed NBN regression with unemployment and emergency admission rates (a) overall (b) acute (c) chronic; stratified by age group adjusting for baseline ethnicity and proportion of population with chronic disease. Model 2 as per model 1 additionally adjusting for proxies of access to primary and secondary care.<br>Sensitivity analysis:

	1. Using mixed-effects models 1 to 2 rather than fixed effects models (authors should explain why this approach used)<br>2. Repeating fixed effects Model 1 using longer cohort (2012-2019)<br>3. Repeating Models 1 to 2 using different definition of ACSC<br><br>Please consider or report measures of model fit.<br><br>Results<br><br>The results section should state the percentage of medium output areas for which data are available.<br><br>Interpretation of the results should be left to the discussion section to avoid overstating the reliability of the measures of access to care.<br><br>A narrative on unemployment prevalence during the study period is important for interpreting the significance of changes in unemployment rates - as authors present change in admission for a 1% increase in unemployment (mean unemployment is 1% at the end point of the study period).<br><br>Discussion<br><br>The discussion should acknowledge the limitations of the measures of access to primary care and the lack of data on community-based care (a key driver of vaccination rates for example).<br><br>The increase in admission rates despite decreasing unemployment rates suggests other factors are driving the increase, which should be discussed.<br><br>The limitations section should be expanded to include a discussion on the limitations of access to health service data and the lack of measures of community-based care.<br><br>Supplementary Materials<br><br>The supplementary materials should be better integrated into the main text, with clear signposting to relevant figures and tables and better care over clear labelling and formatting of tables.

VERSION 1 – AUTHOR RESPONSE

Reviewer: 1

• We'd first like to thank you for the opportunity to review this paper. It is a cleverly constructed study with the particularly nice feature of capturing a temporal measure of socioeconomic conditions. The paper itself is clear and well-written, and we're glad to see this work being done. We think this submission is suitable for BMJ Paediatrics.

Response: Thank you very much for your kind comments.

• Abstract:

• Pulling sentence one from the introduction into the abstract could aid readers less familiar with ACSCs.

Response: Thank you. This has been amended.

• Abstract: The conclusion states the main effect was not explained by differential access to care - this is perhaps a little too strongly worded given how hard it is to measure access to care, and due to the strength of the measure for secondary care access used.

Response: Thank you, this has been amended as outlined in the marked copy, as recommendation in a subsequent reviewer's comment:

"Increasing trends in neighbourhood unemployment were associated with increases in paediatric emergency admission rates for ACSCs in England. This was not explained by available measures of differential access to care, suggesting policy interventions should address the causes of unemployment and poverty in addition to health system factors to reduce emergency admissions for ACSCs."

• Intro:

• Suggest a clarifying statement about the fact that ACSCs, avoidable admissions, preventable hospitalisations, avoidable hospitalisations have been used as interchangeable concepts in the literature to mean hospitalisations which could have been avoided with timely effective outpatient (not necessarily primary) care. The distinction is important as, although it's hard to pin down an agreed definition, for children, diabetes and epilepsy are rarely managed in primary care so the one currently presented may be confusing to readers.

• Suggest clarifying note that some are unavoidable e.g. severe asthma exacerbations

Response: Thank you, the lack of a clear definition can be problematic and should be further highlighted. We have expanded our definition to include "primary or outpatient care", and have amended the following:

"Ambulatory care sensitive conditions (ACSCs), are those conditions for which hospital admission could be prevented by interventions primary or outpatient care[1]. ACSCs, avoidable admissions, preventable hospitalisations, and avoidable hospitalisations have been used interchangeably in the literature to mean hospitalisations which could have been avoided with timely effective outpatient care (not necessarily primary care) [2]."

Please also find further amendments in the discussion (final statement underlined below):

"This is the first large scale longitudinal analysis, using routinely available data and different methods to adjust for potential confounders and mediators, showing an association between increasing neighbourhood unemployment and emergency hospital admission rates for children in England with ACSCs. We build confidence in the causality between SEC and ACS hospitalisations by using the best available time-varying measure of SECs, measured at MSOA level and therefore a reasonable proxy for individual SECs. Whilst we cannot explicitly distinguish potentially avoidable admissions

without individual case notes, our careful use of the defined ACSCs attempts to provide the best available indicator of ambulatory care [41].”

- Methods:

• Clarification requested as to whether this exposure is the only area-level time-varying proxy for SEC at all in the UK, or if it is the only one publicly available, the only one from the sources available to the authors, etc.

Response: It is the only (annual) area-level time-varying proxy for SEC at all in the UK. This has been further clarified (underlined):

“The primary exposure variable was the annual neighbourhood unemployment rate, defined as the percentage of people aged 16–64 years in each neighbourhood who were claiming Jobseeker’s Allowance or Universal Credit principally for the reason of being unemployed, provided by the Place-based Longitudinal Data Resource (PLDR)[26, 27]. This time-varying measure of small-area deprivation is the only measure of SECs at all that is available annually for neighbourhoods in England, and we treat it as a proxy for childhood SECs [28], in the absence of other suitable time-varying measures of SECs available at the neighbourhood level that would allow us to examine trends and relationships over time. Annual population estimates from the ONS were used to derive denominators for the outcome and exposure [29].”

We further clarify this again in the limitations “We use the only annual proxy measure of SECs; however, unemployment may not be the strongest indicator of neighbourhood SECs.”

- The measures for access to primary care are quite nice in combination. Concerns that at MSOA level there may be a lot of variation, and also as to who responds to the GPPS surveys. If they are known to be representative, a line to give that reassurance would be really nice. The secondary care measure is much weaker, however, we appreciate this is hard to capture and it is still a nice addition as a secondary analysis, as long as the conclusions drawn are not too strong.

Response: Thank you, this should be discussed further. This is now added to the limitations subsection.

“Whilst we attempt to capture access to primary and secondary care by using openly available proxy measures, there are also limitations to these measures. MSOA-level measures may introduce increased variation across these measures, which could be mitigated using smaller area estimates. Whilst weighted GPPS data improves the representativeness, explicit demographic information on the responders to the surveys is unknown. A cautious approach towards conclusions of the mediating effect from these measures is therefore adopted. Further research using additional proxy measures (such as community-based care measures) would improve interpretations drawn.”

- It does not seem that access to care is necessarily strictly a mediator, and as it has only been controlled for, not subject to mediation analysis (as far as we can see in the paper), some additional justification here could be useful. It seems plausible that poor access to care leads to higher unemployment as well as more ACSC admissions, and that some access issues are less problematic if you are not currently employed, etc.

Response: Thanks. Clarification on the approach used within this study (Specifically, not using mediation approaches) has now been given. As per other reviewers’ comments, the limitations subsection has also been expanded to consider issues like the one you raise here (see below).

- The text states “we additionally adjusted for access to care measures, to evaluate whether these were likely to be mediating”. We think this is a little unclear as if we are assessing whether they are mediators, a mediation analysis would be better. If we’re simply trying to isolate the main effect, then this analysis is OK. Further clarity on the methods used may be sufficient to address all the comments on mediation.

Response: Thank you for these comments. Careful consideration surrounding discussion around mediation has now been made across the manuscript. Specifically in the limitations:

In limitations: “Some limitations should be considered. Suppression due to small count numbers at MSOA level limited data to two-year intervals, and broad age stratification. There is a possibility of ecological bias within this study, where associations found at the group level are not present at the individual level. These data do not account for repeat admissions, and we are unable to quantify how this may influence results. We use the only annual proxy measure of SECs; however; unemployment may not be the strongest indicator of neighbourhood SECs. Whilst we attempt to capture access to primary and secondary care by using openly available proxy measures, there are also limitations to these measures, and future research would benefit from investigating other access to health service data that may become available. MSOA-level measures may introduce increased variation across these measures, which could be mitigated using smaller area estimates. Whilst weighted GPPS data improves the representativeness, explicit demographic information on the responders to the surveys is unknown. A cautious approach towards conclusions of the mediating effect from these measures is therefore adopted. Further research using additional proxy measures (such as community-based care measures) would improve interpretations drawn. Finally, this study does not use robust methods for exploring mediation, and so conclusions drawn on the mediating effect of variables used in this analysis are limited [43]. A more robust approach for exploring mediation would be beneficial in future research to help mitigate barriers to inferring causality (such as a possible bidirectional relationship between SEC and the number of ACSC hospitalisations).”

- Results:

• Tables are to a different degree of precision to the text (3.dp vs 2.dp).

Response: Thank you for raising this. The main text has been amended to 3.dp.

• P-values and standard * approach available in supplementary tables, but not in the main manuscript. Is there a reason for not including the *? CIs helpful, but nice to get a simpler indication that results are comfortably significant without having to check the supplementary materials.

Response: Thanks, we have now included the * approach within the main manuscript.

- Discussion: The line that states “we overcome the challenges faced by earlier studies to inferring causality…” is too strong. This is a much nicer design to build confidence in the causality, but we do not believe it is fair to say the challenges have been overcome. There are still barriers to inferring causality e.g., strength of the proxy for SEC, a risk of endogeneity through unmeasured confounders/mediators and a potential for a bidirectional relationship between SEC and the number of ACSC hospitalisations, etc.

Response: Thank you for raising this. The paragraph you refer to in the discussion has been amended as follows:

“This is the first large scale longitudinal analysis, using routinely available data and different methods to adjust for potential confounders and mediators, showing an association between increasing neighbourhood unemployment and emergency hospital admission rates for children in England with ACSCs. We build confidence in the causality between SEC and ACS hospitalisations by using the best available time-varying measure of SECs, measured at MSOA level and therefore a reasonable proxy for individual SECs.”

Further, the limitations have been more clearly addressed, which can be found above in previous responses.

• Discussion: Though the statement is accurate and fair, we think including ‘Conservative-led’ risks politicising the paper in a way that could detract from the impartiality of the important conclusions it draws.

Response: Thank you, this has been amended.

“The stronger effect of neighbourhood unemployment on ACS admissions between 2012-2017 could be potentially explained by the lag effect of austerity measures implemented by the government from 2010, with their full impact on communities becoming more evident over time.”

• Discussion: ‘may give better causal estimates’ would be preferable to ‘may give the best causal estimates’, even if within the context of this study, they may be the best.

Response: Thank you, this has been amended.

• Discussion: The null result (ACS Chronic, 10-19Yr olds) is restated but not explored in any detail the discussion. Not a necessity, but some discussion of this would be a nice addition.

Response: Thank you, the following has been added:

“However, there was no observed association in chronic ACSCs for older children (10-19 years). This may be indicative of the differential impact of socioeconomic stressors (such as unemployment changes) on children of different ages. For example, younger children may have increased parental dependency and may be more vulnerable to unemployment changes that directly affect their caregivers. No association was observed for chronic ACSCs in both ages over a longer period, further suggesting that economic hardship may have a more immediate impact on acute health needs. For example, chronic ACSCs require more long-term care with the impact of these conditions manifesting over an extended period. This may create more long-standing relationships with health services, which could mitigate some immediate effects of changes in SECs.”

• Discussion: Typo – ‘Theses data do not account for…’

Response: Thank you, this has been amended.

Reviewer: 2

Summary

This ecological study examines the relationship between neighbourhood deprivation, measured by unemployment rates, and hospital/emergency admission rates for ambulatory care sensitive conditions (ACSC) in children aged 0-19 years over a five-year period (2012-2017) in England. Understanding the link between poverty, health, and health service use is crucial, and this analysis aims to quantify elements of these relationships.

Key findings include

Emergency admission rates increased over time, particularly for acute ACSC (reported over 2008-2019). However, the main analysis period (2012-2017) shows little variation in acute ACSC admission rates and an increase in chronic ACSC.

Neighbourhood unemployment rates decreased from approximately 3.5% to 1% during 2012-2017, though this is not detailed in the results.

Emergency admission rates were associated with unemployment rates for all groups except children aged 10-19 with chronic ACSC. A 1% change in unemployment rates corresponded to a ~3% increase in ACSC admission rates, equivalent to a doubling of unemployment rates by the end of the period.

Adjustments for primary and secondary care access proxies did not alter these associations.

In the main regression analyses, unemployment rates were positively associated with admission rates, while the time period was negatively associated. Adjustments for baseline ethnicity and prevalence of long-term conditions did not impact these associations, nor did proxies for access to care. These associations were most evident in the 0-9 years age group. Sensitivity analyses had minimal impact on the associations.

The study makes a valuable contribution to understanding the impact of socio-economic status on hospital use. However, significant revisions are needed, particularly in the methods and results sections, to better describe the work.

Strengths and Importance

The study addresses a significant public health issue by exploring the impact of socio-economic factors on health outcomes in children. The use of multiple datasets (HES, ONS, PLDR, GPPS, and the 2011 Census) across most of England provides a comprehensive approach to understanding these relationships. Delineating acute v chronic conditions is also a helpful approach to potentially understanding drivers of potentially avoidable health care use.

Response: Thank you for your comments and clear summary. This is very helpful.

- Areas for Improvement

While the topic is important, the methods and results sections could benefit from clearer presentation to enhance understanding.

Abstract

The abstract effectively highlights the study's focus but could be more balanced in discussing the impact of emergency admissions. For instance, while ACS emergency admissions can strain the NHS and be traumatic for children, they can also resolve underlying health issues.

Response: Thanks. Whilst the abstract is limited by the word count, the introduction has ensured this point is considered.

"While emergency admissions for ACSCs can sometime resolve underlying health issues for individuals, collectively they also put significant pressures on the NHS, such as delays in treatment for other patients [9] , and have been estimated to cost the NHS £1.42 billion annually[10]. Children admitted to hospital are also at risk of infections, medical errors, drug reactions and emotional trauma[11, 12]."

- Abstract: The conclusion in the abstract might overstate the findings. A more accurate reflection could be: "This was not explained by available measures of differential access to care, suggesting policy interventions should address the causes of unemployment and poverty in addition to health system factors to reduce emergency admissions for ACSCs."

Response: Thank you, this has been amended as suggested.

- Introduction: It would be helpful to describe the proportion of emergency admissions that are ACSC versus non-ACSC to provide context.

Response: Thanks, we have now provided further information in the introduction to reflect this:

"UK based studies have reported between 11-40% of emergency department (ED) attendances are non-urgent, or 'inappropriate'[2, 3]. Prior to the COVID pandemic, emergency admissions for ACSCs increased by 18% between 2008-2020[4]. These rates continue to steadily increase[5, 6], with the

proportion of avoidable admissions rising faster than the overall rate of emergency admissions (a 14% increase of emergency admissions for conditions that might be manageable in primary care, compared to a 9.3% increase for all types of emergency admissions) [7].”

• Methods: No additional analysis recommended, merely tighter clearer description and reporting of analysis. The methods section needs more clarity. It is not fully clear whether the authors are examining ED attendance or hospital admissions. Clarifying this distinction is crucial.

Response: Thanks for raising this. We have clarified the definition of emergency admissions and standardised the text throughout.

“Emergency admissions (emergency hospital inpatient stays) for ACSCs were identified from Hospital Episodes Statistics (HES) data.”

• Methods: A narrative around the DAG should be included in the main text.

Response: Thank you, this has been included at the end of the Methods section:

“The logic model (supplementary figure S1) describes the hypothetical causal pathway between income deprivation and emergency hospital admissions for paediatric ACSCs, and the potential effects of the covariates we had data for. Between-area differences in unmeasured variables that remain constant over the study period are accounted for automatically in fixed-effects models. A full description of the measures and data sources can be found in the Supplementary file (Table S1).”

• Methods: A detailed description of the datasets and the conditions included in the acute and chronic categories should be provided in the main text.

Response: Thanks, please find the following amendment:

The primary data were obtained from Hospital Episode Statistics (HES), a data warehouse that contains details of clinical, patient, administrative and geographical information about an individual patient admitted to an NHS hospital in England. The primary outcome of interest was emergency admissions (emergency hospital inpatient stays) for ACSCs. We split this outcome into three outcome variables based on ICD-10 codes: ED admissions with a primary diagnosis of (1) an acute ACSC (Vaccine-preventable diseases, Lower respiratory tract infections, Upper respiratory tract infections, Dehydration and gastroenteritis, Urinary tract infections); (2) a chronic ACSC (Asthma, Diabetes, Epilepsy); and (3) any ACSC (all ACS conditions). This approach to defining ACSCs has been used elsewhere [27]. ICD-10 codes are detailed in supplementary file (Table S24). We derived biennial emergency admissions for each neighbourhood in England, from 2012-2017. Due to suppression of data, annual data were not available.”

• Methods: The primary outcome of interest should be clearly stated, and the rationale for using biennial admission rates instead of annual rates should be explained.

Response: Thanks, this has now been addressed, as per the response above.

•   Methods: More information on the data and measures used would be beneficial. For example, a simple statement outlining the datasets and their specific uses could improve clarity.

Response: Thanks. Considering this point raised, and other comments previously, please find the following in the methods section (underlined):

"The primary data were obtained from Hospital Episode Statistics (HES), a data warehouse that contains details of clinical, patient, administrative and geographical information about an individual patient admitted to an NHS hospital in England. The primary outcome of interest was emergency admissions (emergency hospital inpatient stays) for ACSCs. We split this outcome into three outcome variables based on ICD-10 codes: ED admissions with a primary diagnosis of (1) an acute ACSC (Vaccine-preventable diseases, Lower respiratory tract infections, Upper respiratory tract infections, Dehydration and gastroenteritis, Urinary tract infections); (2) a chronic ACSC (Asthma, Diabetes, Epilepsy); and (3) any ACSC (all ACS conditions). This approach to defining ACSCs has been used elsewhere [27]. ICD-10 codes are detailed in supplementary file (Table S24). We derived biennial emergency admissions for each neighbourhood in England, from 2012-2017. Due to suppression of data, annual data were not available.

To control for access to care, several indicators were provided by PLDR [28]. We used time-varying measures of primary care access (the number of general practitioners (GPs) per capita serving the population (derived from NHS Digital data)[32], and the proportion of the population who describe their experience of making a GP appointment as poor (derived from General Practice Patient Survey [GPPS] data)[33]; and secondary care access (the average road network distance to the nearest hospital with an ED [34]. These have previously been used as proxy measures for access to care [30].

Time-invariant baseline measures of ethnic composition, and the age-specific prevalence of long-term health problems per neighbourhood, derived from 2011 Census[26], were included in robustness tests (mixed-effects models) to account for potential confounding. There is evidence to show they are related to both deprivation and the hospitalisation outcomes[8, 27, 35, 36]."

•   Methods: Regression models need to be more clearly described and outcome clarified (is the main outcome the delta variation in admission rates and proportion?) e.g. main analysis: "Model 1 fixed NBN regression with unemployment and emergency admission rates (a) overall (b) acute (c) chronic; stratified by age group adjusting for baseline ethnicity and proportion of population with chronic disease. Model 2 as per model 1 additionally adjusting for proxies of access to primary and secondary care.

Sensitivity analysis:

1. Using mixed-effects models 1 to 2 rather than fixed effects models (authors should explain why this approach used)

2. Repeating fixed effects Model 1 using longer cohort (2012-2019)

3. Repeating Models 1 to 2 using different definition of ACSC

Response: Thank you, this has been more clearly described.

Main analysis: "We used fixed-effects negative binomial regression models to investigate the association between annual changes in unemployment and changes in ACS emergency admissions between neighbourhoods, holding constant unobserved time-invariant characteristics for each

neighbourhood. We fitted two models between 2012 and 2017 (restricted by data availability). Model 1 fixed negative binomial regression with unemployment and emergency admission rates (a) overall (b) acute (c) chronic ACSCs; stratified by age group adjusting for all unmeasured time-invariant confounding factors. Model 2 as per Model 1 additionally adjusting for proxies of access to primary and secondary care. The number of emergency admissions were modelled using the log of the population as an 'offset' variable, indicating the maximum number of admissions that could have occurred. Likelihood ratio tests supported the inclusion of a quadratic time-trend. The models estimate the IRR, which is interpreted as the percentage change in the incident rate of emergency admissions associated with the changes in neighbourhood unemployment rate. The outcome was stratified by age group (0-9, 10-19 years) to account for different rates of hospital admissions related to ACSCs in children of different ages. Analyses were performed using R [version 4.3.0]."

Sensitivity analyses: "First, models 1 and 2 were repeated (2012-2017) using mixed-effects negative binomial regression. Mixed effects models included a random intercept to account for the longitudinal nature of the data (i.e. observations clustering over time). These investigate the effect of unemployment changes whilst allowing for individual differences between neighbourhoods in baseline admission rates, and are used as a robustness test to determine whether between-neighbourhood variation impacts the findings from the main analysis. This alternative method was used to assess the relationship between deprivation trends within neighbourhoods and trends in paediatric ACS emergency admission rates. Mixed effects models allow for direct adjustment of time-invariant confounders. We therefore stratified by age group adjusting for baseline ethnicity and proportion of population with chronic disease. Second, fixed effects model 1 (without mediators) was fitted between 2008-2019 to investigate the association between changes in neighbourhood unemployment and admission rates for ACSCs over a longer time-period. We fitted our primary models over shorter time frame from 2012-2017 because of the availability of mediator data over this period, but were able to repeat our main fixed-effects analysis, without mediators, over a longer period as a sensitivity analysis. Third, to investigate the appropriateness of our chosen definition of ACSCs, we fitted models 1 and 2 using a narrower definition of ACSCs (using ICD-10 codes described in supplementary file) that was derived from existing literature[37]."

- Please consider or report measures of model fit.

Response: Thank you, these have been added to the models within the supplementary file

- Results: The results section should state the percentage of medium output areas for which data are available.

Response: Thanks, this has been included:

"A total of 6,771 (99.7%) English neighbourhoods had available outcome data from 2008-2019."

- Results: Interpretation of the results should be left to the discussion section to avoid overstating the reliability of the measures of access to care.

Response: Thank you. Any interpretation (e.g. "indicating main effects were not explained (mediated) by differential access to care") within the results has been removed and kept only for discussion subsection.

•	A narrative on unemployment prevalence during the study period is important for interpreting the significance of changes in unemployment rates - as authors present change in admission for a 1% increase in unemployment (mean unemployment is 1% at the end point of the study period).

Response: Thanks, please now find the relevant information and the end of the paragraph within the results subsection:

“A total of 6,771 (98.7%) English neighbourhoods had available outcome data from 2008-2019. Figure 1 shows emergency admission rates, per 100,000, for English neighbourhoods by age group for acute, chronic, and all ACSCs, 2008-2019. There were considerably more admissions for acute ACSCs than chronic ACSCs. Younger children accounted for more admissions than older children. For acute ACSCs, average admission rates rose in younger children to 2736 per 100,000 between 2008 and 2019, an overall increase of 10%. For older children, rates increased by 37%, with an average admission rate of 444 per 100,000 in 2019. For chronic ACSCs, average admission rates decreased by 27% in younger children, and 3% in older children, over time. Unemployment prevalence decreased from 2.2% in 2012 to 1.4% in 2017 (see supplementary figure S2 for biennial unemployment prevalence.”

•	Discussion: The discussion should acknowledge the limitations of the measures of access to primary care and the lack of data on community-based care (a key driver of vaccination rates for example).

Response: Thank you. As per previous reviewer’s comments, the limitations section now includes the following:

“We use the only annual proxy measure of SECs; however, unemployment may not be the strongest indicator of neighbourhood SECs. Whilst we attempt to capture access to primary and secondary care by using openly available proxy measures, there are also limitations to these measures, and future research would benefit from investigating other access to health service data that may become available. MSOA level measures may introduce increased variation across these measures, which could be mitigated using smaller area estimates. Whilst weighted GPPS data improves the representativeness, explicit demographic information on the responders to the surveys is unknown. A cautious approach towards conclusions of the mediating effect from these measures is therefore adopted. Further research using additional proxy measures (such as community-based care measures) would improve interpretations drawn. Finally, this study does not use robust methods for exploring mediation, and so conclusions drawn on the mediating effect of variables used in this analysis are limited [43]. A more robust approach for exploring mediation would be beneficial in future research to help mitigate barriers to inferring causality (such as a possible bidirectional relationship between SEC and the number of ACSC hospitalisations).”

•	Discussion: The increase in admission rates despite decreasing unemployment rates suggests other factors are driving the increase, which should be discussed.

Response: Thank you, this has now been discussed in the discussion subsection:

“We support previous findings of a social gradient in ACSCs ED admission[13, 19, 22], and showed that neighbourhood unemployment, used here as a proxy for childhood socio-economic conditions [30], was a main predictor for increased avoidable emergency admissions. We provide evidence that increasing trends in unemployment is associated with increasing trends in emergency admissions for all ACSCs in children. This association was found despite an observed rise in admission and falling unemployment rates. However, this may be explained by the lagged effects of unemployment on

health, for example the compounding of the social determinants of health across vulnerable neighbourhoods. Further, these trends in unemployment are overall trends and may not reflect trends at the individual MSOA level ”

• Discussion: The limitations section should be expanded to include a discussion on the limitations of access to health service data and the lack of measures of community-based care.

Response: Thanks, please see point above regarding relevant amendments to limitations subsection.

• Supplementary Materials: The supplementary materials should be better integrated into the main text, with clear signposting to relevant figures and tables and better care over clear labelling and formatting of tables.

Response: Thank you, this has now been actioned across the manuscript.

VERSION 2 – REVIEW

**REVIEWER NAME**	***Ingrid Wolfe***
**REVIEWER AFFILIATION**	King's College London, Women's and Children's Health
**REVIEWER CONFLICT OF INTEREST**	
**DATE REVIEW RETURNED**	14-Dec-2024

**GENERAL COMMENTS**	Thank you for the opportunity to see the revised manuscript; i am content.

**REVIEWER NAME**	***Michelle Heys***
**REVIEWER AFFILIATION**	University College London Institute of Child Health
**REVIEWER CONFLICT OF INTEREST**	Not applicable
**DATE REVIEW RETURNED**	28-Nov-2024

**GENERAL COMMENTS**	Thank-you for responding to the reviewer comments.
